# Alterations in mosquito behaviour by malaria parasites: potential impact on force of infection

**DOI:** 10.1186/1475-2875-13-164

**Published:** 2014-05-01

**Authors:** Lauren J Cator, Penelope A Lynch, Matthew B Thomas, Andrew F Read

**Affiliations:** 1 Department of Life Sciences, Imperial College London, Ascot, UK; 2 Mathematics and Statistics Department, The Open University, Milton Keynes, UK; 3 Center for Infectious Disease Dynamics, Department of Entomology, Pennsylvania State University, University Park, PA, USA

**Keywords:** Manipulation, Malaria, Transmission, Model, Vector-parasite interactions

## Abstract

**Background:**

A variety of studies have reported that malaria parasites alter the behaviour of mosquitoes. These behavioural alterations likely increase transmission because they reduce the risk of vector death during parasite development and increase biting after parasites become infectious.

**Methods:**

A mathematical model is used to investigate the potential impact of these behavioural alterations on the lifetime number of infectious bites delivered. The model is used to explore the importance of assumptions about the magnitude and distribution of mortality as well as the importance of extrinsic incubation period and gonotrophic cycle length. Additionally, the model is applied to four datasets taken from actual transmission settings.

**Results:**

The impact of behavioural changes on the relative number of lifetime bites is highly dependent on assumptions about the distribution of mortality over the mosquito-feeding cycle. Even using fairly conservative estimates of these parameters and field collected data, the model outputs suggest that altered feeding could easily cause a doubling in the force of infection.

**Conclusions:**

Infection-induced behavioural alterations have their greatest impact on the lifetime number of infectious bites in environments with high feeding-related adult mortality and many pre-infectious feeding cycles. Interventions that increase feeding-associated mortality are predicted to amplify the relative fitness benefits and hence enhance the strength of selection for behavioural alteration.

## Background

Infection with malaria parasites has been shown to alter the behaviour of mosquitoes, with effects varying depending on parasite life stage [1]. When a female mosquito ingests malaria parasites from a human host, it is not immediately able to transmit the infection onto a new host. This is because the parasite must go through several developmental stages before becoming infectious. During this pre-infectious period, female mosquitoes are less attracted to hosts [2] and less persistent in their feeding attempts [3]. When they do feed, these females probe less frequently and for shorter durations than uninfected females [4-7]. After development in the mosquito midgut, which typically lasts ten to fourteen days in high transmission settings [8,9], the parasites move into the haemolymph and eventually to the salivary glands, at which point the mosquito is able to infect a new vertebrate host. Infectious females have been reported to be more attracted to hosts [2,10], more persistent in feeding attempts [3], feed on more hosts per feeding attempt [11], probe more frequently [4-7], and suffer greater feeding-associated mortality [12] than uninfected females.

This suite of behavioural changes associated with infection seem likely to increase parasite transmission (fitness) and so has been interpreted as adaptive manipulation of host behaviour by the parasite [1]. However, the observed changes might equally be host adaptations, or non-adaptive side effects of infection (pathology) [2,13]. Whatever their cause, the behavioural alterations have potential to impact transmission. Here, the likely magnitude of this impact was analysed not least because the behaviour of infected mosquitoes has been largely ignored in models of malaria epidemiology to date [1].

In a previous publication, the effect of behavioural alteration on the number of infectious bites a female would be predicted to deliver in its lifetime was briefly described by a simple mathematical model [1]. This model measured the impact of two of the most commonly reported behavioural manifestations of malaria infection, namely, decreased feeding in the oocyst stage and multiple biting/feeding per gonotrophic cycle in the sporozoite stage. Here, for the first time, the derivation of the model is presented, the importance of different assumptions about how mosquito mortality is distributed across the gonotrophic cycle is further investigated, and the model is applied to a suite of data from real-world transmission sites to determine the potential impact of behavioural alteration on malaria transmission dynamics in a variety of ecological contexts.

## Methods

### Model

**Figure 1:**
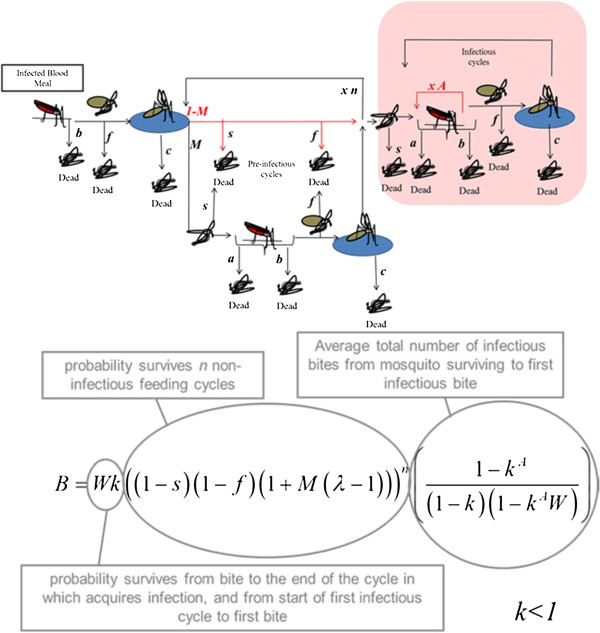
**Mathematical model used to determine the effect of altered feeding behaviour on the relative force of infection.** Schematic of the model components that are used to calculate the average number of infectious bites delivered per infected female in its lifetime (*B*). See text for symbol definitions.

A mathematical model was used to investigate the potential impact of behavioural alteration on the relative force of infection, which is defined here as the average number of infectious bites delivered per infected female. All females enter this model after taking and being infected by an infectious blood meal. During each gonotrophic cycle mosquitoes search for hosts, blood feed, rest, search for oviposition sites, and oviposit. These cycles take place over a set period of days and are repeated throughout the mosquito’s adult life until death (Figure 1).

As the females go through these cycles they experience two potential sources of mortality (Figure 1). Background mortality is defined as death occurring at a constant daily rate, independent of the activity undertaken by the mosquito. It is the product of mortality applicable over the time period assigned to host-seeking pre-bite (*s*) and the time spent resting and seeking an oviposition site post-bite (*f*). Incremental mortality associated with each bite is also assumed. This is divided between mortality, which occurs immediately prior to biting, (*a*) (preventing the bite and transmission of parasites) and mortality immediately after biting (*b*), and with each oviposition attempt (*c*). In the case of unaltered behaviour, all females surviving long enough to do so will seek a blood meal in every gonotrophic cycle, take one bite, and subsequently lay eggs. The model assumes that mosquito population size is unaffected by any change in mosquito fecundity arising from behavioural alteration.

The average number of infectious bites an infected female will deliver in its lifetime (*B*) is calculated as:

$$B=\mathrm{Wk}{\left(\left(1-s\right)\left(1-f\right)\left(1+M\left(\lambda -1\right)\right)\right)}^{n}\left(\frac{1-k^{A}}{\left(1-k\right)\left(1-k^{A}W\right)}\right)$$

Pre-infectious cycles are those in which females have been infected with malaria parasites that have not yet developed into transmissible stages. The probability of surviving a pre-infectious cycle is calculated as the probability of surviving background mortality during the cycle, multiplied by the probability of either not feeding (1-*M*) or feeding (*M*) and surviving the associated feed and oviposition (*λ*), with *λ* = (1-*c*)(1-*a*)(1-*b*).

The probability of surviving the total incremental mortality associated with one bite, *k*, is defined as (1-*a*)(1-*b*). The probability of surviving from one blood feed to the next, *W*, is calculated as *W* = (1-*f*)(1-*c*)(1-*s*). The number of gonotrophic cycles between the cycle in which a malaria infection is acquired and the first cycle in which the mosquito can give an infectious bite is represented by *n*.

To account for alterations in behaviour associated with infection, the probability of females attempting to take a blood meal in the pre-infectious (oocyst) stage (*M*) and the number of bites per cycle when infectious with sporozoites (*A*) was adjusted. Thus, (1-*M*), the probability that a female does not feed during a pre-infectious feeding cycle, represents pre-infectious changes in feeding behaviour. This phenotype is based on work suggesting that females in this stage are less persistent and less likely to attempt to feed in this period [2-4]. Similarly, the parameter *A* expresses the changes in feeding behaviour associated with the infectious or sporozoite-stage in which females are more likely to give multiple bites during each feeding attempt [11].

Females that do not bite in a given cycle are still assumed to experience background mortality for one cycle, but not the incremental mortality arising from biting and laying eggs. Females taking multiple bites during one feeding attempt are assumed to experience the incremental bite-related mortalities, *a* and *b,* for each attempted bite. The relative number of lifetime infectious bites is calculated by comparing the average number of infectious bites per infected female in the altered case with that in the unaltered case, where the average number of infectious bites per infected female is a product of the probability of surviving to the transmissible stage of infection and the average number of infectious bites per mosquito reaching this stage (Figure 1).

If malaria parasites do not alter mosquito behaviour, *M* = 1 (females will take a blood meal during all pre-infectious cycles) and *A* = 1 (each female only bites once per feeding attempt), so that the average number of infectious bites delivered by a female in its lifetime, *B*_0_, is

$$\;B_{0}=\frac{\mathrm{Wk}{\left(\left(1-s\right)\left(1-f\right)\right)}^{n}\lambda^{n}\left(1-k\right)}{\left(1-k\right)\left(1-\mathrm{kW}\right)}$$

If malaria parasites do alter host behaviour, then *M* < 1, and *A* > 1. The impact of behavioural alteration on force of infection was calculated as *F*, the proportionate increase in lifetime infectious bites between the behaviourally altered (*B*) and unaltered case (*B*_*0*_).

*F* is calculated as

$$F=\frac{B}{B_{0}}=\frac{{\left(1+M\left(\lambda -1\right)\right)}^{n}\left(1-k^{A}\right)\left(1-\mathrm{kW}\right)}{\lambda^{n}\left(1-k\right)\left(1-k^{A}W\right)}$$

A detailed derivation of the model is given in Additional file 1.

When considering the maximal effect of behavioural alterations *M* was set to 0 (all females skipping pre-infectious feeds) and *A* = 5 (5 bites per infectious feeding attempt). Sporozoites can be transmitted during probing and prior to blood ingestion [14], so this represents the number of attempted bites rather than the number of completed blood meals per feeding event. Given that there are already data showing that infected females can take at least two bites per attempt [11] and that in some circumstances even uninfected females have been found to ingest blood from up to three hosts per night [15,16] this seems a biologically plausible upper value for *A*. For our analysis these represent the maximal behavioural alteration infection might create. Using these parameters allows for comparison of the maximum potential effects altered feeding could create under different conditions.

### Mortality distributions across the gonotrophic cycle

There are few data on mosquito mortality in the field. Those data that do exist come from methods such as mark-release recapture and comparing ratios of infection stages, Christopher stages and parity rates [8,17-20]. These methods provide information about the averaged mortality per feeding cycle, but do not describe the distribution of that mortality over a feeding cycle. There is evidence that the distribution of mortality may be heavily associated with feeding events. Mosha and others [21] reported feeding-associated mortality as high as 25.5% in the absence of any mosquito control intervention. To capture these uncertainties, three different distributions are used here to describe mortality over a gonotrophic cycle. In the first, mortality is evenly distributed throughout the feeding cycle. In the second, the mortality is completely associated with feeding events and the total mortality reported for a cycle is split evenly between mortality incurred immediately before and after biting. The third distribution assumes that females die at a constant daily rate and experience additional mortality (evenly split pre-and post-bite). Taking a commonly used daily mortality of 15% (which applied consecutively over three days gives a cumulative mortality of 38.6% per feeding cycle) [9,22], these three mortality scenarios are as given in Table 1.

**Table 1:** Generalized mortality distributions

**Distribution**	***a***	***b***	***c***	***s***	***f***	**Daily**	**Total**
**Background**	0.00%	0.00%	0.00%	21.64%	21.64%	15.00%	38.59%
**Feeding-associated**	21.64%	21.64%	0.00%	0.00%	0.00%	0.00%	38.59%
**50% of each**	11.48%	11.48%	0.00%	11.48%	11.48%	7.811%	38.59%

Three distributions of mortality were used in the model: mortality evenly distributed over a feeding cycle, mortality completely associated with feeding attempts and an intermediate scenario in which mortality half of the mortality was evenly dispersed over a feeding cycle and half of the total mortality was associated with the feeding event.

Symbols: *a* refers to pre-bite mortality, *b* refers to post-bite mortality, Total, is the mortality over a cycle.

### Field data sets

Killeen and others describe a suite of transmission-related parameters for four malaria-endemic locations [9] (Table 2). Butelgut, is located in a forested inland region of the Madang Province in Papua New Guinea [20]. The primary vector is *Anopheles punctulatus* and there was transmission of both *Plasmodium falciparum* and *Plasmodium vivax* during the study period [23]. The remaining sites are African. Kankiya and Kaduna are both dry savannah sites in northern Nigeria and at the time the data were collected had holo-endemic *P. falciparum* transmission [18,24]. Namawala is in the flat flood plains of the Kilombero Valley in Tanzania [25]. *Anopheles gambiae sensu lato* is the primary vector in Namawala [25] and Kaduna [18], while transmission in Kankiya is dominated by *Anopheles arabiensis*[24]. The model was run using the conditions described in these transmission settings to demonstrate the potential impact of behavioural alteration in field settings.

**Table 2:** Parameters used for the four endemic-malaria sites

	**Gonotrophic cycle length/days**	**# Pre-infectious cycles**	**Total mortality per feeding cycle**	**Daily mortality**	**Pre-bite mortality**	**Post-bite mortality**
**Butelgut**	3.7	2	42.8%			
All-daily				14.0%	0	0
All-feeding				0	24.4%	24.4%
**Kankiya**	3	3	17.0%			
All-daily				6.0%	0	0
All-feeding				0	8.9%	8.9%
**Namawala**	2.7	4	39.6%			
All-daily				17.0%	0	0
All-feeding				0	22.25%	22.25%
**Kaduna**	2	5	19.0%			
All-daily				10.0%	0	0
All-feeding				0	10.0%	10.0%

First three data columns, published estimates. Second three data columns, mortality values estimated from measured feeding cycle mortality rates, allocated across the feeding cycle according to the mortality scenarios described above (Table 1). For more information about these data sets please see [9].

## Results

### Effect of behavioural alterations in a generic case

First, the model is used to investigate the potential impact of manipulation using a set of generic parameters that are commonly used to model transmission [22]. The gonotrophic cycle is assumed to last three days [26] and each blood meal leads to the completion of a gontrophic cycle (there was no gonotrophic discordance). There are four feeding cycles (12 days) between the female taking an infectious blood meal and becoming infectious [27]. The number of times an altered female bites per feeding episode is limited to five. Using these assumptions, situations are compared where daily mortality is constant across the gonotrophic cycle, is explicitly linked to feeding, or is a combination of both (Table 1).

**Figure 2:**
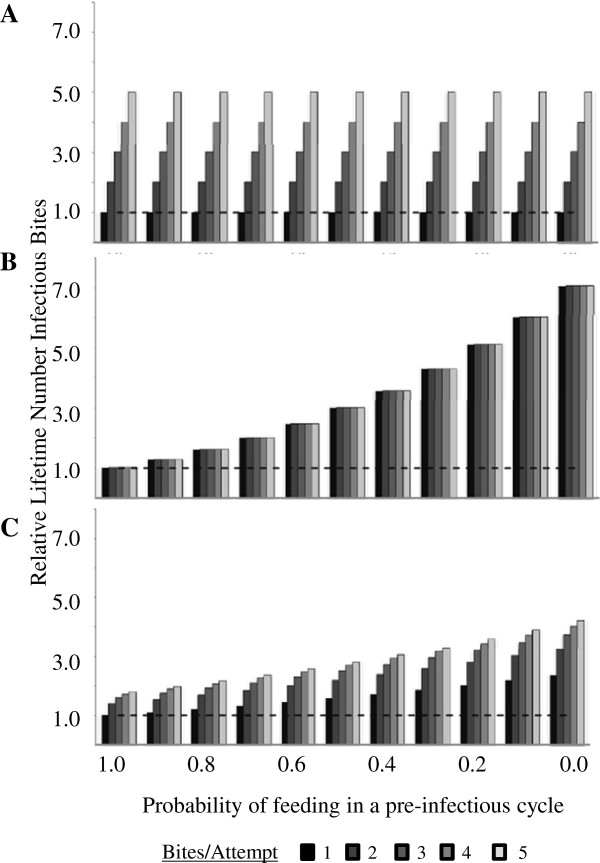
**Increase in lifetime infectious bites due to behavioural alterations (altered feeding propensity before and after infectiousness). A.** Mortality constant across a feeding cycle; **B.** All mortality associated with feeding; **C.** Mortality split equality between those two scenarios. The different shaded bars from left to right indicate one to five post-infectious bites. The y-axis is proportionate increase, so the dotted line denotes the situation where behavioural alteration has no impact on transmission.

When mortality is evenly distributed across the gonotrophic cycle and not associated with a feeding event, there is no survival cost to females attempting multiple bites during a single feeding attempt (*A* > 1). Therefore, these females increase the relative number of lifetime infectious bites per female by one with each additional bite for a maximal five-fold increase (Figure 2A). There is no increase in the number of infectious bites per infected female from pre-infectious manipulation in this scenario because manipulated females are not more likely to survive by skipping feeding, and the associated mortality, in pre-infectious cycles.

Alternatively, if mortality is entirely related to feeding, there is no mortality associated with the time spent between feeding attempts and so females will take bites until the mortality associated with a bite leads to their death. Therefore, females will deliver the same average number of infectious bites in their lifetime regardless of whether those bites are distributed five per feeding attempt or one per feeding attempt. With this mortality assumption an unaltered female delivering one bite per feeding attempt will survive through more feeding cycles to deliver the same number of bites as an altered female taking five bites per feed (no difference between bars in a cluster, Figure 2B). However, females skipping pre-infectious feeds reduce feeding-associated mortality and hence experience a large relative increase in survivorship (increased *F* from left to right, Figure 2B). Maximal impact on transmission occurs when all females skip pre-infectious cycles. These alterations in behaviour lead to up to seven-fold increases in transmission intensity.

In reality, mortality is likely associated with both feeding and day-to-day events. When half the mortality risk is associated with a feeding event and the remainder distributed across daily (background) mortality, the relative increase in the number of lifetime infectious bites as a result of behavioural alteration is intermediate between the two extreme mortality scenarios (compare Figure 2C with A,B).

### Influence of oviposition related mortality

Next, the effect of oviposition-related mortality on impact of behavioural alteration on transmission is investigated (Table 3). There are no data on oviposition-related mortality in the field, but it is frequently observed in the laboratory. If there is significant oviposition-associated mortality in the field, it would substantially increase the relative force of infection resulting from behavioural alteration. This can be seen by considering the extreme case, where all mortality is related to oviposition and that *M* = 0 and A = *5* (the maximum behavioural alterations possible in the model). All oviposition-related mortality is avoided if pre-infectious feeds are skipped (since mosquitoes which do not feed, do not oviposit) resulting in a seven-fold increase in transmission intensity. Additionally, with this mortality assumption, a female incurs no additional mortality irrespective of how many bites it takes in order to secure one full blood meal, all mortality being incurred as oviposition-associated mortality when it goes to lay the resulting clutch of eggs. This allows an additional five-fold increase in transmission intensity. This leads to a 35-fold (seven-fold by five-fold) increase in transmission (Table 3).

**Table 3:** The effect of different mortality assumptions on the relative increase in force of infection

**Distribution**	**Daily**	***a***	***b***	***c***	**Total**	**Max *F***
Background	15%	0%	0%	0%	38.6%	5
Feeding- associated	0%	21.6%	21.6%	0%	38.6%	7
Oviposition	0%	0%	0%	38.6%	38.6%	35

Mortality distributions were calculated based on the parameters of the generalized case (15% daily mortality, three-day gonotrophic cycle, and four pre-infectious cycles). Details of each mortality distribution are also given. Note that in the all-feeding mortality scenario, that feeding-associated mortality is evenly split between immediately pre-and post-bite.

Symbols; *a* refers to pre-bite mortality, *b* refers to post-bite mortality, and *c* refers to ovipostion-related mortality, Total is the mortality over the cycle. The relative increase in the number of infectious bites per female (Max *F*) is reported for a scenario in which all manipulated females skip pre-infectious feeding cycles (*M = 0*) and infectious females take five bites per feeding attempt (*A = 5*).

### Potential impacts of behavioural alteration in transmission settings

The reported gonotrophic cycle length, number of cycles the female completed during sporogony and daily survivorship reported for the four data settings [9] were applied to the contrasting mortality allocations as described in the generic case above (Table 1) to explore the predicted effects on transmission. Recall again, that *F* is a relative measurement and so while the lifetime number of infectious bites per female at each of the four sites differs, the relative increases in *F* within each site under varying degrees of infection-induced behavioural alteration is reported.

**Figure 3:**
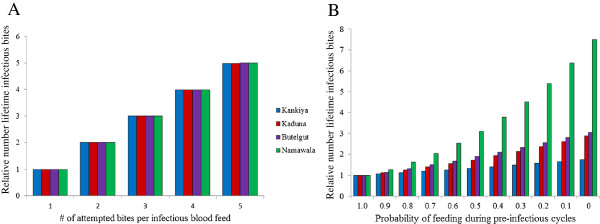
**The predicted effect of behavioural alteration on transmission at four different sites.** Y-axis is the relative lifetime number of infectious bites per female (*F*) and each transmission site represented by different coloured bars. **A*.****F was* calculated assuming constant daily mortality. In this scenario, relative increases in *F* are driven by the number of attempted bites per infectious feed *(A)*. *F* is a relative measure within sites and so the relative increases do not vary between sites under this mortality assumption, even though the absolute magnitude of transmission intensity varies among the sites. **B.** Values generated assuming all mortality is feeding-related. In this instance there is no effect of the number infectious bites on *F* (see Figure 2) and thus, the effect of the probability of feeding during pre-infectious feeds (1-M) is displayed.

With mortality assumed to be constant and not explicitly linked to feeding, the relative effects of altered behaviour are equivalent across sites, yielding up to a five-fold increase in lifetime number of infectious bites depending on the number of bites per feed (Figure 3A). In contrast, if mortality is linked to feeding, the background parameter values lead to large variations between sites (Figure 3B). In the Kankiya field site, the relative increase in force of infection based on pre-infectious (oocyst) stage alteration is negligible even if mosquitoes skip all pre-infectious feeds. For the Namawala site, on the other hand, skipping feeds has a progressively large (>seven-fold) effect on relative force of infection because the combined effect of both relatively high mortality rate and high number of pre-infectious feeds leads to a greater survival “pay-off” in this setting when females skip feeds and avoid the associated mortality.

### Influence of pre-infectious cycles

As described in the generalized case, the relative increase in force of infection arising from a given behavioural modification is highly sensitive to the per cycle mortality and also to the number of pre-infectious cycles. For example, at a site with a daily mortality of 19%, a three-day gonotrophic cycle, and four pre-infectious cycles, the maximum relative increase in lifetime infectious bites (assuming all mortality is feeding-related) is 12-fold.

To further investigate the relative importance of gonotrophic cycle length and the number of pre-infectious cycles on the relative impact of behavioural alteration on lifetime infectious bites, the case where mortality rates were split evenly over daily and feeding-associated mortality are considered. Parameter values are used from the standardized case, the Kankiya site that has relatively low mortality per feeding cycle (17% per feeding cycle), and the Namawala site that has relatively high mortality (39% per feeding cycle). The duration of gonotrophic cycles are adjusted without changing the number of pre-infectious cycles (standard = 4, Kankiya = 3, Namawala = 4) to look at the effect of changing cycle duration. To look at the effect of the number of pre-infectious cycles, the cycle durations are maintained (standard = 3, Kankiya = 3, Namawala = 2.7) and the number of pre-infectious cycles are adjusted.

**Figure 4:**
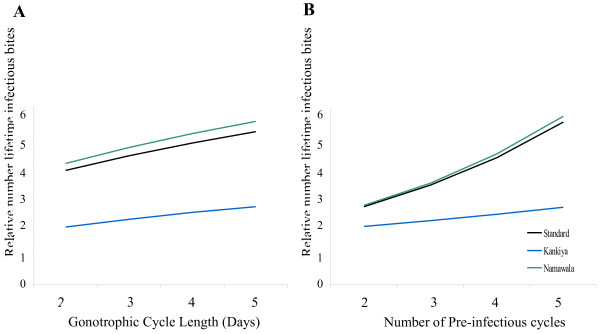
**The effect of gonotrophic cycle length and number of pre-infectious cycles on relative lifetime number of infectious bites per female resulting from manipulation.** In both graphs, the values reported are based on transmission site data, each line represents a location (associated daily mortality value) with an even split between daily and feeding-associated mortality. One value was held constant while the other was varied (Tables 1 and 2) The relative lifetime number of infectious bites reported is for a scenario in which all manipulated females skip pre-infectious feeding cycles (*M = 1*) and they take five bites per infectious feeding attempt (*A = 5*). **A.** the number of pre-infectious cycles was held constant and the duration of gonotrophic cycles was altered. **B.** Effect of the number of pre-infectious cycles by holding the cycle duration constant and varying the number of pre-infectious cycles.

While longer cycle length and higher numbers of pre-infectious cycles increase the relative survival pay-off from behavioural alteration, the number of pre-infectious cycles in which the female skips a blood meal has a bigger impact on the effects of behavioural changes than the length of the cycle. The pay-off is only slightly changed in a setting like Kankiya, but is more dramatically affected in Namawala, where the mortality per feeding cycle is higher and adjusting cycles from two to five results in a doubling of relative force of infection (Figure 4). This is the case when a portion of the total mortality is assumed to be associated with the feeding event and would not be true if the mortality were wholly associated with daily background mortality.

### Minimum manipulation required for large impacts on *F*

**Figure 5:**
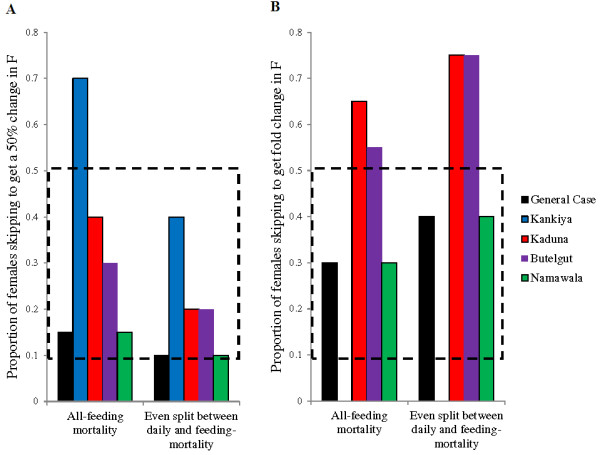
**Minimum proportion of females skipping pre-infectious feeds (1-*M*) required to cause large increases in the force of infection, *F*.** The minimum proportion of females required to skip pre-infectious feeds (1-*M* in the model) was calculated. Infectious females were assumed to attempt only two bites per feed (*A* = 2). Parameter values and mortality distributions are as defined in Tables 1 and 2. **A.** The proportion of females required to skip pre-infectious feeds to achieve a 50% increase in *F*. **B.** The proportion of skipping females required to cause a fold increase in *F.* The dashed lines represent the range of females reported to skip pre-infectious bloodmeals, (1-*M*), in laboratory studies [2-4,7,10]. Under most transmission parameters the required proportion of females skipping pre-infectious blood meals in order to cause large increases in the force of infection falls well within the range observed in laboratory studies. Note that in Kankiya there is no proportion of females at which *F = 2.* This site has very low averaged daily mortality compared to the other transmission settings. Even if 100% of females skip all pre-infections feeds the increase in the relative force of infection is less than 100% when *A* is capped at 2 (*F* = 2 never reached).

While useful for comparing the potential impact of different parameters on force of infection, one could argue that the maximal conditions in the model (*M* = 0 and *A* = 5) are unrealistically extreme. The model was run for all parameter sets using the three mortality distributions and a conservative assumption that altered females take only one additional infectious bite (*A = 2*). Using these assumptions, the minimum proportion of females required to skip pre-infectious bloodmeals (1-*M*) in order to achieve a 50% increase and a doubling in the relative force of infection was calculated (Figure 5). Even this conservative assumption about infectious biting rate, combined with field parameters, our analysis predicts that behavioural alteration within a laboratory derived range for *M*[2-4,7,10,11] would cause at least a 50% increase in force of infection in most transmission settings.

## Discussion

The analysis presented here indicates that depending on baseline transmission ecology and the strength of the behavioural alteration, infection-induced behavioural alterations could impact the number of infectious bites per infected mosquito by many fold. Highly plausible parameter combinations quickly double the force of infection and some parameter space gives changes of three- to seven-fold. To put these figures into perspective, the basic reproductive rate, *R*_*0*_, scales directly with *F*, the measure of the potential impact of behavioural alteration (for further explanation of this relationship see Additional file 2). Under certain parameter conditions and assumptions the presence or absence of behavioural modification can make a seven-fold difference to the lifetime number of infectious bites given by infectious mosquitoes. This means that estimates of *R*_*0*_ from models which assume infected mosquitoes behave as uninfected mosquitoes could be many fold off.

Consider the magnitude of public health interventions on transmission intensity. A recent study in the Kilombero Valley found a 4.2-fold decrease in the number of infectious bites per person per night (Entomological Inoculation Rate) associated with the use of bed nets and an additional 4.6-fold decrease with the additional use of long-lasting, insecticide-treated nets [28]. Thus, in certain environments, the impact of behavioural alteration on calculated metrics is potentially of the same magnitude as widespread control measures. Again, this does depend on mortality distribution and the prevalence and intensity of altered phenotypes, but even using conservative assumptions, behavioural alteration has the potential to be very important. Behavioural alteration is thus relevant both for the theoretical analyses which underlie public health policy decisions and for the derivation of parameters values indirectly derived from accessible field data. That such a potentially important phenomenon is so poorly understood and is ignored in most current theoretical frameworks represents a serious knowledge gap [11].

A key unknown is the magnitude of the behavioural alterations (Figures 2 and 4). The proportion of total females in a population that exhibit pre-infectious behavioural alterations (1-*M*, the x-axis in Figures 2 and 3) has not been estimated in the field. In laboratory studies, with age-matched controls, females have been found to be 11-50% less likely to attempt to feed [2,4] and 20% less persistent when they do [3]. More is known about feeding propensity of females after they become infectious (*A*, the different shaded bars in Figures 2 and 3). In the laboratory, sporozoite-infected females were found to be 19-400% more attracted to hosts [2,4,10] than age-matched controls and 23% more persistent [3]. In the field, Koella and others [11] measured a 12% increase in multiple blood meals from sporozoite-infected females and Wekesa and others [7] reported a 43.19% increase in the likelihood of sporozoite-infected females to feed. These two studies, however, were necessarily not conducted with matched controls (as they were from field-caught mosquitoes) and so the increase could be confounded by factors such as age. If manipulation has a similar effect in wild populations, then sporozoite-stage manipulation would lead to a greater increase in relative force of infection than oocyst-stage manipulation using the conditions studied here. However in situations in which mortality is high, increased survivorship in pre-infectious cycles becomes a more important driver of relative force of infection as in the Namawala scenario. The uncertainties here emphasize the need for better behavioural data from the field.

The impact of behavioural alteration on transmission is sensitive to estimates of mortality rates and assumptions of the distribution of mortality across feeding cycles. This approach does not incorporate age-dependent mortality in which older females are subject to higher mortality than younger females. If older females experience higher background mortality, this underlying mortality pattern would be expected to increase the relative importance of early infectious bites and reduce the importance of changes in the number of attempted bites per feed.

Feeding-associated mortality was an important determinant of the impact of behavioural alteration. Therefore, the relative “pay-off” of infection-associated behavioural changes is likely to interact with mosquito control strategies that modulate vector mortality. For example, in experimental hut tests, Mosha and others [21] found that bed nets treated with deltamethrin and α-cypermethrin caused an approximate 50% feeding-associated mortality per night in *An. arabiensis*. This was compared with the mortality of 25% in control groups. The relative increase in force of infection was calculated for these mortalities assuming that all mortality is associated with feeding. In the case without bed nets (25% mortality) maximum behavioural alteration causes a three-fold increase in relative force of infection. When feeding-associated mortality is increased to 50% (as with bed nets) alteration results in an almost 16-fold increase in relative force of infection. This increase in the relative force of infection is due to pre-infectious females avoiding the additional mortality associated with feeding. Thus, interventions that increase mortality (especially feeding-associated mortality) will magnify the impact of these infection-associated behavioural phenotypes on transmission intensity. In other words, if behavioural alteration is widespread, it could be reducing the control efficacy of a number of widespread public health interventions in ways that are not currently captured by the theoretical frameworks available to evaluate them.

If infection-induced alteration of mosquito-feeding behaviour is parasite manipulation (i e, the result of parasite adaptation), the strength of natural selection for pre-infectious manipulation would be expected to increase as interventions increase feeding-associated mortality (because the fitness pay-off for skipping pre-infectious cycles rises). This may make little difference if manipulation is cost-free for the malaria parasites, since selection will already have generated the maximum manipulation possible. However, in many instances manipulation can be costly to parasites [29]. For instance, if manipulation is achieved by the secretion of costly levels of behaviour-altering hormones or if resources allocated to manipulation effort trade-off with parasite replication rates, then natural selection will favour the level of manipulation which balances those costs and benefits. The widespread use of control measures such as bed nets would then increase the costs associated with feeding and therefore, the benefits of manipulation, which would lead to the evolution of more manipulative malaria parasites. Similar evolution might also be expected if behavioural alteration is an adaptive response by the mosquitoes to infection. In this scenario, females exhibit altered phenotypes due to a cost or constraint associated with feeding in a pre-infectious cycle. Insecticide-driven selection would reinforce the benefits of skipping gonotrophic cycles in the pre-infectious phase.

## Conclusion

The model analysis suggests that alterations of mosquito-feeding behaviour following infection have the potential to greatly impact malaria transmission in natural settings, and to affect the efficacy of vector control interventions. The analysis also makes clear that further progress requires a much better quantitative characterization of behavioural alteration in the field, and of the patterns of mortality associated with blood feeding and oviposition across the mosquito lifespan. The model provides a framework to assess these data if and when they become available. A refined understanding of the causes and consequences of behavioural alteration in the field could lead to novel approaches for controlling malaria by targeting manipulated phenotypes.

## Competing interests

The authors declare that they have no competing interests.

## Authors’ contributions

PL led model design with input from LJC; LJC and PL ran model analyses with input from MBT and AFR; LJC, PL, AFR, and MBT wrote the manuscript. All authors read and approved the final manuscript.

## Supplementary Material

Additional file 1 **Mathematical derivation of the relative number of infectious bites predicted with behavioural alteration (*F*).** Click here for file

Additional file 2 **Relationship between model output parameter *F* to basic equation for R**_**0**_**.** Click here for file
