# Supplementary material for: Alterations in mosquito behaviour by malaria parasites: potential impact on force of infection
Source: Malar J. 2014 May 1;13:164. doi: 10.1186/1475-2875-13-164 (PMC4113138; doi:10.1186/1475-2875-13-164)
Supplement: Additional file 1 — Mathematical derivation of the relative number of infectious bites predicted with behavioural alteration ( F ). [file 1475-2875-13-164-S1.pdf]

Additional File 1

Description	symbol
Time spent host seeking pre-bite	s
Time spent resting and seeking an ovipositing site post-bite	f
Feeding-related mortality pre-bite	a
Feeding-related mortality post-bite	b
Oviposition-related mortality	c
Probability that infected mosquito will feed (and lay) in pre-infectious cycle	M
Probability of survival during the time between blood feeds	W
Probability of surviving one feeding and one ovipositing event	λ
Number of feeding cycles between infection and infectiousness	n
Number of bites taken per feeding attempt for infectious mosquitoes	A
Probability of surviving single bite	k
Average lifetime number of infectious bites given per infected mosquito	B
Average lifetime infectious bites with no behavioural alteration	B_0
Number of infectious bites with behavioral alteration as proportion of that without	F

$$k = (1-a)(1-b)$$

$$\lambda = (1-c)k$$

$$W = (1-s)(1-f)(1-c)$$

Probability that a newly infected mosquito survives from the bite in which it acquires *plasmodium* infection to first post-infection feeding attempt
$= (1-b)(1-s)(1-f)(1-c) = (1-b)W$

Probability of surviving from one bite to the next $= (1-a)(1-b)(1-s)(1-f)(1-c)$

Probability of surviving period of one feeding cycle if does not feed or lay
$= (1-s)(1-f)$

Probability of surviving period equivalent to one post-infection, pre-infectious feeding cycle
$= (1-s)(1-f)\big(M(1-a)(1-b)(1-c) + (1-M)\big) = (1-s)(1-f)(M\lambda + 1 - M)$
$= (1-s)(1-f)\big(1 + M(\lambda - 1)\big)$

Average number of bites per feeding attempt for mosquito which survives to give one bite and attempts A bites
$= 1 + (1-b)(1-a) + \big((1-b)(1-a)\big)^2 + \big((1-b)(1-a)\big)^3 .... \big((1-b)(1-a)\big)^{A-1}$

Using the standard sum for a finite series this can be simplified to

$$=\frac{1-\left(\left(1-b\right)\left(1-a\right)\right)^{A}}{1-\left(1-b\right)\left(1-a\right)}=\frac{1-k^{A}}{1-k}$$

The average number of post-infectious feeding attempts, each comprising A attempted bites, from a mosquito which survives to give a first infectious bite

$$=1+\left(\left(1-b\right)\left(1-a\right)\right)^{A-1}\left(1-b\right)\left(1-s\right)\left(1-f\right)\left(1-c\right)\left(1-a\right)+\left(\left(1-s\right)\left(1-f\right)\left(1-c\right)\left(\left(1-a\right)\left(1-b\right)\right)^{A}\right)^{2}$$
$$+\left(\left(1-s\right)\left(1-f\right)\left(1-c\right)\left(\left(1-a\right)\left(1-b\right)\right)^{A}\right)^{3}.....+\left(\left(1-s\right)\left(1-f\right)\left(1-c\right)\left(\left(1-a\right)\left(1-b\right)\right)^{A}\right)^{\infty}$$

$$=1+Wk^{A}+\left(Wk^{A}\right)^{2}+\left(Wk^{A}\right)^{3}....\left(Wk^{A}\right)^{\infty}$$

Using the standard sum of an infinite series, this can be simplified to

$$\frac{1}{1-Wk^{A}}$$

Average lifetime number of infectious bites per infected mosquito

$=$ probability survives to end of feeding cycle in which infected
$\times$ probability survives n infected feeding cycles
$\times$ probability survives from end of pre-infectious feeding cycles to give first infectious bite
$\times$ average number of feeding attempts per mosquito which survives to give first infectious bite
$\times$ average number of infectious bites per feeding attempt

probability survives to end of feeding cycle in which infected $=\left(1-b\right)W$

probability survives n infected feeding cycles $=\left(\left(1-s\right)\left(1-f\right)\left(1+M\left(\lambda-1\right)\right)\right)^{n}$

probability survives from end of last pre-infectious feeding cycle to give first infectious bite $=\left(1-a\right)$

average number of feeding attempts per mosquito which survives to give first infectious bite $=\dfrac{1}{1-Wk^{A}}$

average number of infectious bites per feeding attempt $=\dfrac{1-k^{A}}{1-k}$

So, B, the average lifetime number of infectious bites per infected mosquito

$$= \left(1-b\right)W\left(\left(1-s\right)\left(1-f\right)\left(1+M\left(\lambda-1\right)\right)\right)^{n}\left(1-a\right)\frac{1}{1-Wk^{A}}\frac{1-k^{A}}{1-k}$$

$$B = Wk\left(\left(1-s\right)\left(1-f\right)\left(1+M\left(\lambda-1\right)\right)\right)^{n}\frac{1-k^{A}}{\left(1-k\right)\left(1-Wk^{A}\right)}$$

B_0, the average lifetime number of infectious bites per infected mosquito in the absence of behavioural modification =

$$= \left(1-b\right)W\left(\left(1-s\right)\left(1-f\right)\left(1+1\left(\lambda-1\right)\right)\right)^{n}\left(1-a\right)\frac{1}{1-Wk^{1}}\frac{1-k^{1}}{1-k}$$

$$B_{0} = Wk\left(\left(1-s\right)\left(1-f\right)\lambda\right)^{n}\frac{1-k}{\left(1-k\right)\left(1-Wk\right)}$$

So the number of infectious bites per infected mosquito with behavioral manipulation, as a proportion of that without manipulation is given by

$$F = \frac{Wk\left(\left(1-s\right)\left(1-f\right)\left(1+M\left(\lambda-1\right)\right)\right)^{n}\frac{1-k^{A}}{\left(1-k\right)\left(1-Wk^{A}\right)}}{Wk\left(\left(1-s\right)\left(1-f\right)\lambda\right)^{n}\frac{1-k}{\left(1-k\right)\left(1-Wk\right)}}$$

$$F = \frac{\left(1+M\left(\lambda-1\right)\right)^{n}\left(1-Wk\right)\left(1-k^{A}\right)}{\lambda^{n}\left(1-Wk^{A}\right)\left(1-k\right)}$$
