# Supplementary material for: Alterations in mosquito behaviour by malaria parasites: potential impact on force of infection
Source: Malar J. 2014 May 1;13:164. doi: 10.1186/1475-2875-13-164 (PMC4113138; doi:10.1186/1475-2875-13-164)
Supplement: Additional file 2 — Relationship between model output parameter F to basic equation for R 0 . [file 1475-2875-13-164-S2.pdf]

Additional File 2

Consider the basic equation for R_0, adapted to reflect low human mortality rate relative to the latent period[1]

$$R_0 = \frac{ma^2bc}{\mu_2\gamma} e^{-\mu_2\tau_2}$$

m = number of susceptible mosquitoes
a = biting rate per mosquito
c = proportion of bites producing infection in mosquito
μ_2 = mosquito mortality rate
b = proportion of bites from infectious mosquitoes which generate infection in humans
γ = infectious period in human host
τ_2 = latent period in mosquito

$$R_0 = \frac{mabc}{\gamma}\left(\frac{a}{\mu_2} e^{-\mu_2\tau_2}\right)$$

$e^{-\mu_2\tau_2}$ = probability of mosquito surviving latent period

$a\frac{1}{\mu_2}e^{-\mu_2\tau_2}$ = number of infectious bites per infected mosquito

Our manipulation metric '*F*' is the proportionate difference in the number of infectious bites per infected mosquito if specific behavioural modifications are taken into account, versus that assuming no behavioral modification. This value can therefore be incorporated directly into R_0 simply by multiplying the number of infectious bites per infected mosquito by *F*, ie. R_0 simply scales with '*F*' value, so R_0 with behavioural modification = R_0F .

---

[1]*Anderson and May, "Infectious Diseases of Humans: Dynamics and Control" publ. Oxford University Press, equation 14.11 adjusted per text page 400 to reflect low human mortality rate relative to latent period*
